# *Wolbachia* in the Flesh: Symbiont Intensities in Germ-Line and Somatic Tissues Challenge the Conventional View of *Wolbachia* Transmission Routes

**DOI:** 10.1371/journal.pone.0095122

**Published:** 2014-07-02

**Authors:** Crystal L. Frost, Steven W. Pollock, Judith E. Smith, William O. H. Hughes

**Affiliations:** 1 Institute of Integrative Biology, University of Liverpool, Liverpool, United Kingdom; 2 Department of Biology, University of York, York, United Kingdom; 3 School of Environment and Life Sciences, University of Salford, Salford, United Kingdom; 4 School of Life Sciences, University of Sussex, Brighton, United Kingdom; International Atomic Energy Agency, Austria

## Abstract

Symbionts can substantially affect the evolution and ecology of their hosts. The investigation of the tissue-specific distribution of symbionts (tissue tropism) can provide important insight into host-symbiont interactions. Among other things, it can help to discern the importance of specific transmission routes and potential phenotypic effects. The intracellular bacterial symbiont *Wolbachia* has been described as the greatest ever panzootic, due to the wide array of arthropods that it infects. Being primarily vertically transmitted, it is expected that the transmission of *Wolbachia* would be enhanced by focusing infection in the reproductive tissues. In social insect hosts, this tropism would logically extend to reproductive rather than sterile castes, since the latter constitute a dead-end for vertically transmission. Here, we show that *Wolbachia* are not focused on reproductive tissues of eusocial insects, and that non-reproductive tissues of queens and workers of the ant *Acromyrmex echinatior*, harbour substantial infections. In particular, the comparatively high intensities of *Wolbachia* in the haemolymph, fat body, and faeces, suggest potential for horizontal transmission via parasitoids and the faecal-oral route, or a role for *Wolbachia* modulating the immune response of this host. It may be that somatic tissues and castes are not the evolutionary dead-end for *Wolbachia* that is commonly thought.

## Introduction

Symbionts can greatly affect the evolution, ecology and behaviour of their hosts [1]. This affect is dependent on the specific phenotypic effect, the transmission route and tissue-tropism of the symbiont, all of which interact [2]–[4]. Specialisation for particular transmission routes is seen in many symbionts. This is often manifest by infections being focussed on specific tissues that are integral to the transmission route. For example, malaria enhances transmission to humans by infecting mosquito salivary glands [5], infections of the faecal-orally transmitted microsporidian *Nosema apis* are confined to the midgut of its honeybee hosts [6], and certain serovars of the sexually transmitted *Chlamydia trachomatis* specifically infect the epithelial cells of the genital tract [7]. Within the *Rickettsia*, transovarial vertical transmission can be high, but horizontally transmission to mammals from their tick hosts is enhanced by increased replication of the bacteria in the midgut epithelia [8]. The investigation of tissue-specific symbiont densities could therefore provide valuable insights into the transmission route utilised.

*Wolbachia* has been described as the greatest ever panzootic, infecting many insect species as well as a wide diversity of other arthropods and filarial nematodes [9], [10]. It is an obligate intracellular endosymbiont which appears to be highly adapted for maternal vertical transmission. In filarial nematodes this specialisation has led to a mutualism forming whereby *Wolbachia* is integral to the reproduction of the host [11]. In arthropods *Wolbachia* is best known for its female-biased distortion of host sex ratios (e.g. via male killing) which increase vertical transmission or the comparative fitness of infected females [12]–[14]. However, the effects of *Wolbachia* in arthropods are now known to be more diverse, ranging from a beneficial influence on host survival and resistance to other parasites, to detrimental effects that can even cause host death [14], [15]. Additionally phylogenetic analysis of host co-association provides evidence that there is also significant horizontal transmission of *Wolbachia* over evolutionary timescales [16]–[18]. This is further corroborated by experimental studies of *Wolbachia* transmission in parasitoids wasps of *Drosophila* and Lepidoptera which show that parasitoids can gain new infections from both infected hosts and from other parasitoids with which they share a host [19]–[21]. The relative importance of vertical and horizontal transmission for *Wolbachia* in these hosts is therefore unclear. The wide spectrum of symbiont-induced phenotypic effects and transmission strategies in one bacterial lineage makes *Wolbachia* an excellent model for investigating the evolution of such traits.

As a vertically transmitted reproductive parasite, we would predict that *Wolbachia* would predominantly infect the ovaries, however in a number of species *Wolbachia* has also been isolated from other tissues such as the fat body and gut [3], [4], [22]–[26]. Indeed, the results from two studies demonstrate that infection intensities in non-reproductive tissues can be considerable [4], [25]. One particularly interesting group in respect to the transmission, tissue tropism and effects of *Wolbachia* are the eusocial insects. These species are by definition split into reproductive queen and more-or-less sterile worker castes, making the colony a ‘superorganism’, with the castes comparable to the germ-line and somatic tissues within a single individual [27]. ‘Somatic’ castes, as well as tissues, would be a dead-end for vertical transmission. Furthermore, any negative effects of parasite infection on the worker caste may reduce the overall production of future queens by a colony and in turn the vertical transmission of *Wolbachia*. *Wolbachia* may thus be selected to be lost from workers, in order to increase the overall success of vertical transmission. Experimental evidence of such a phenomenon comes from two ant species, *Acromyrmex echinatior* and *Formica truncorum*, in which workers were found to have a lower prevalence of infection than queens [28], [29]. *A. echinatior* also belongs to a tribe of ants in which host-*Wolbachia* phylogenetics suggest horizontal transmission is common over an evolutionary timescale, while inter-study comparisons are suggestive of horizontal transmission being frequent at even an ecological timescale [18], [28], [30]. Excitingly, in one ant species, *Acromyrmex octospinosus*, high extracellular *Wolbachia* loads have been found in worker foreguts, suggesting a potential for mutualism between these hosts and their *Wolbachia* [25].

Here we carry out a comprehensive quantitative comparison of *Wolbachia* distribution in host germ-line and somatic tissues to test whether tissue-tropism provides support for the vertical transmission paradigm of *Wolbachia*. We do this using the leaf-cutting ant *A. echinatior*, thus enabling extension of the comparison, by comparing infection intensities in the reproductive queen and sterile worker castes. Relatively high intensities in reproductive tissues and castes would support specialisation for vertical transmission via reproductive parasitism, while relatively high intensities in non-reproductive tissues and castes could indicate the potential importance of horizontal transmission. High burdens may also signal the presence of fitness effects mediated by direct metabolic burden or through immune functioning of the host.

## Methods

### Ethics statement

Permission to collect and export the ant colonies was provided by the Autorid Nacional del Ambiente (ANAM). The work did not involve endangered or protected species.

*Wolbachia* intensities were quantified in 24 workers (all from the large worker caste) (1.8–2.4 mm head width) and 24 queens (gynes sensu stricto) from each of four colonies of *Acromyrmex echinatior* (Ae298, Ae357, Ae398 and Ae07P4). The colonies were collected in Gamboa, Panama, in 2006–2008 and maintained in the laboratory at 27±2°C and 80±10% RH on privet leaves and rice. For each individual, *Wolbachia* intensities were determined separately for four tissues: hind gut, fat body, haemolymph and either midgut in workers or ovary/midgut sample in queens. Haemolymph was collected using a fine capillary and subsequent tissue dissections were performed in distilled water, with the ovary and gut dissected out of the body with fine forceps and the diffuse fat body taken with a pipette. Queens and workers were mature, of similar age based on cuticle colour [31], and were collected within two days of one another. In addition, the ovary and midgut from a further 24 queens from each of four colonies (Ae357, Ae084, Ae085 and Ae07P4) were dissected and extracted separately to elucidate the contribution each of these tissues made to a combined ovary/midgut sample. Finally, samples of faeces were collected from 24 large workers from each of four colonies (Ae084, Ae357, Ae398 and Ae07P4) by gently squeezing the abdomen or directly from the dissected hind gut with a fine capillary.

Tissues were incubated at 56°C overnight in 100 µl of 5% Chelex 100 (BioRad) suspended in 10 µM Tris buffer with 4 µl of Proteinase K (5 µl/ml) and boiled for 15 min. After spinning down, the DNA extract (supernatant) was cleaned with a Onestep-96 PCR inhibitor removal kit (Zymo Research). *Wolbachia* primers and probes were designed using the ABI custom design service based on widely aligned sequences of the relevant *Wolbachia* CoxA gene and the host assay was designed using Primer3 and is based within 18S rRNA gene [32], [33] (see Table S1). *Wolbachia* was quantified using the comparative Ct method [34], which standardises target genes against an endogenous host gene to control for differing tissue quantities. Calibration curves were determined for a number of *Wolbachia* infected samples including whole ant, pooled ovary and gut as well as a sample created by pooling a random collection of the experimental extractions. Efficiencies were 91–99% for *Wolbachia* and 95–99% for host over 10,000-fold changes in DNA concentration, with Ct values between 16–37 for the host and 23–36.5 for *Wolbachia* assay. All qPCRs were run in triplicate and replicates with high standard deviation (>0.5 Ct) or outside of the calibration range were removed from analysis. Samples with non-concordant replicates were rerun or excluded. Negative controls and positive reference samples were included in each run.

Differences in infection density between tissues and castes were examined using linear mixed effects models (LMERs), in the R package *lme4* [35]. Within these models the individual the tissues came from, the caste and colony were specified as hierarchical random factors (random = ∼1|Colony/Caste/Individual), and caste and tissue were specified as fixed effects. Transformation of *Wolbachia* density was implemented to account for the non-normality and lack of homogeneity of residuals (log(*Wolbachia*+0.5)), which was subsequently checked by visual assessment of plots of residuals. A separate model included faeces, using only data from the workers of the three colonies from which collection was possible (Ae357, Ae398 and Ae07P4). P-values were computed using the likelihood ratio test method for all fixed effects (REML = FALSE, specified to allow comparison between models with different fixed effects), and the contrasted f values within the models were used to infer significant differences between specific tissues within and between caste (contrasts were considered to be significantly different if t>2 or <−2). All statistics were carried out in R 2.15.2 [36].

## Results

**Figure 1:**
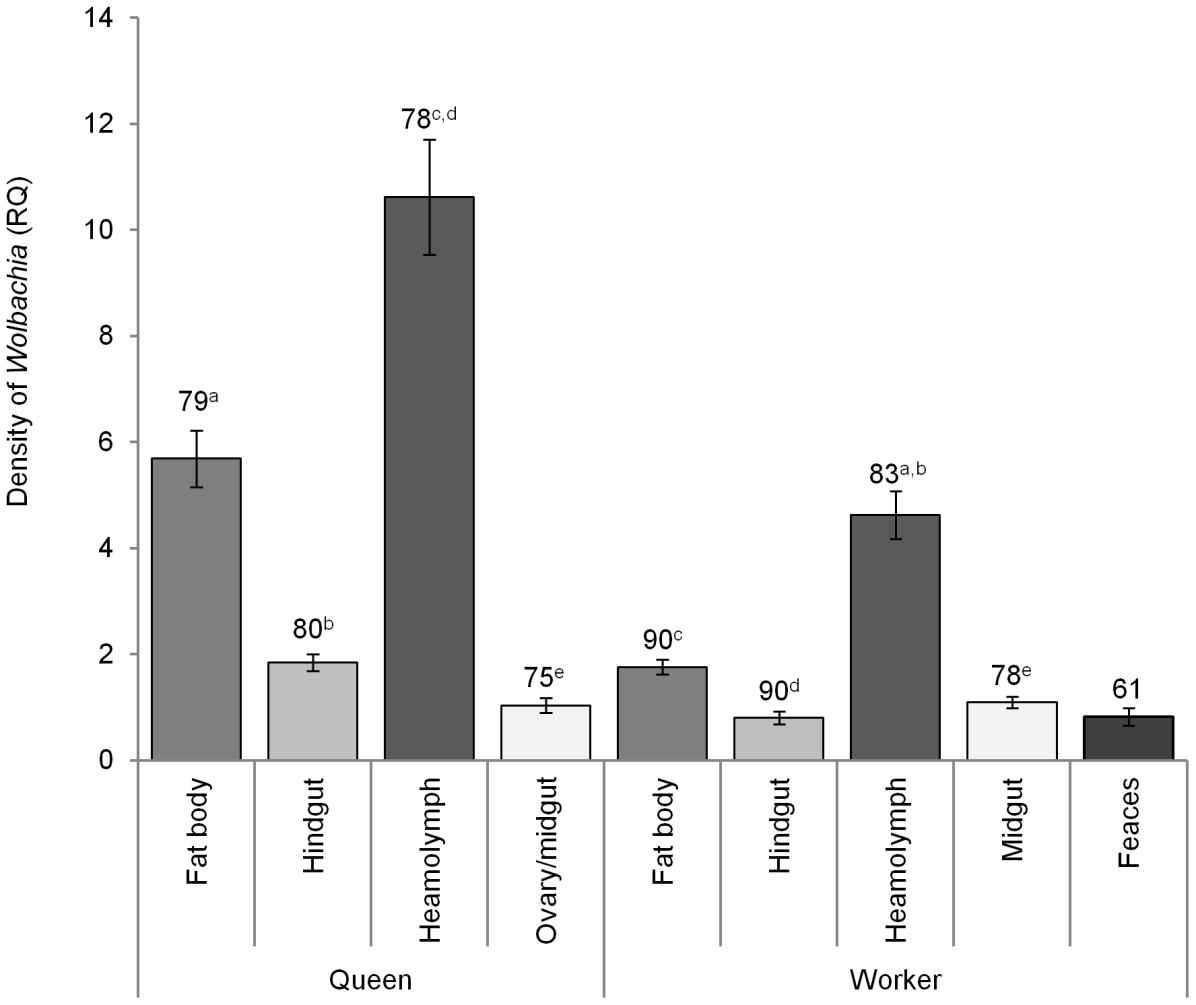
Tissue and caste specific *Wolbachia* infection intensities. Comparison of mean ± s.e. relative *Wolbachia* intensity (RQ) in fat body, hindgut, haemolymph and ovary/midgut of *Acromyrmex echinatior* leaf-cutting ant workers and queens from four colonies, and worker faeces from three colonies. All within-caste contrasts were significant at t<−2 and >2 levels, except for those pairs with the same superscript letters. Sample size is shown above each corresponding bar. Relative *Wolbachia* intensity refers to the intensity of *Wolbachia* normalized against the host 18S control gene.

**Figure 2:**
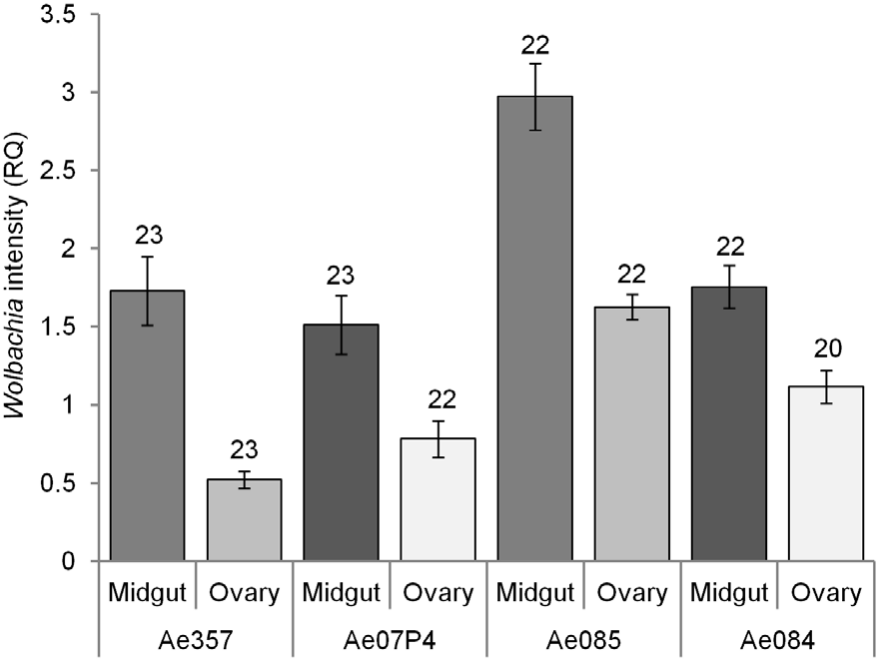
*Wolbachia* infection intensities of the midgut and ovaries of queens. Comparison of mean ± s.e. relative *Wolbachia* intensities (RQ) of the midgut and ovary of *Acromyrmex echinatior* leaf-cutting ant queens from four colonies. Sample size is shown above each corresponding bar. Relative *Wolbachia* intensity refers to the intensity of *Wolbachia* normalized against the host 18S control gene. These intensities are comparable to those in Figure 1.

There was a significant interaction between the effects of tissue and caste on *Wolbachia* intensity (LMER (LRT), df12, 3, χ2 = 90.16, p<0.001). The burden of infection was consistently high in the heamolymph, with almost a five-fold difference between this and the next most burdened tissue in queens and almost three times greater in workers (Figure 1). The fat body also had a significantly high burden of infection in both castes. Workers generally had higher relative intensities of *Wolbachia* in the hindgut than the midgut, with hindgut having a higher *Wolbachia* intensity than the ovary/midgut sample of queens. Overall, queens appeared to have higher intensities, particularly in the fat body, hindgut and haemolymph when compared to their worker counterparts (t = −6.25, t = −4.82, t = −5.42, respectively). While the general patterns were present in all four colonies investigated, the relative abundance of *Wolbachia* in the different tissues differed between colonies, creating a tissue-by-caste-by-colony interaction (LMER (LRT), df36, 21, χ2 = 90.0, p<0.001; Figure S1). Faeces also had significant levels of *Wolbachia*, statistically the same as those in the hindgut and midgut of workers. When analysing the midgut and ovary separately in queens, it was found that intensity was significantly higher in the midgut than in the ovary (LMER (LRT), df5, 1, χ2 = 70.8, p<0.001; Figure 2).

## Discussion

The intensity of *Wolbachia* infections differed significantly between the four tissues and two castes of the leaf-cutting ant, *A. echinatior*. Queens had high levels of *Wolbachia* in the haemolymph and fat body, with intermediate levels being found in the hindgut, and surprisingly, the lowest intensities being found in the ovary. A similar pattern of distribution was seen in the tissues of workers, but with slightly lower intensities than queens for most tissues (fat body, heamolymph and hindgut). We also tested the faeces of worker ants, and found *Wolbachia* at similar levels to those in the hindgut and midgut of this caste.

The observation that levels of *Wolbachia* in the somatic tissues of workers are lower than their queen counterparts suggests that either they regulate infection differently or else that *Wolbachia* growth varies with caste. It has previously been reported that adult workers of this species and of the ant *Formica truncorum*, can have lower infection prevalences than queens and males, suggesting that infection may be lost over a worker’s development [28], [29]. Whilst we did not investigate the infection intensity over time, our results suggest that adult workers maintain infection at a lower level and may therefore have a greater potential to lose infection than queens. Social insect castes are known to differ in immune and hormonal profiles [37]–[42]. The variation in these factors could all provide cues for a parasite-mediated regulation of infection intensity or else affect the way in which the host interacts with different *Wolbachia* strains. It must however be noted that a study of *Acromyrmex octospinosus* has shown that *Wolbachia* infection intensity of workers appears to increase with age [25]. It may be that the increase with age occurs faster in the reproductive queens and this is why higher infection intensity is seen here. Alternatively, it may be that an initial suppression in workers is either absent or at a lower strength in the reproductive females.

The relatively low infection intensity in the ovaries of queens is not consistent with specialisation for reproductive parasitism that has long been thought to be the main transmission strategy of *Wolbachia* [16], [43]. The considerable intensities of *Wolbachia* in somatic tissues of queens and sterile castes found here add to the growing body of evidence that there is far more to *Wolbachia* infections than simply vertical transmission. Here, the distribution of *Wolbachia* seen in the somatic tissues of the queens shows tropism, with higher densities of *Wolbachia* being found in the fat body and haemolymph compared to the gut and ovary, suggesting that the distribution of *Wolbachia* in somatic tissues is not simply a side-effect of infection of the reproductive tissues. There are three non-mutually exclusive hypotheses for this interesting result. First, it could be that horizontal transmission of *Wolbachia* is not an evolutionary ‘accident’ and is instead an important transmission route for *Wolbachia*, as supported by phylogenetic studies [10], [16], [18], [44]. Second, infection of the somatic tissues could be maintained if transfer of *Wolbachia* between somatic and germ tissue is required for vertical transmission. In the ovaries of *Drosophila melanogaster* and *Zyginidia pullula*, for example, *Wolbachia* have been found in high densities in bacteriocyte-like cells that are possibly of somatic origin [45]. The authors of this study note that the vertically transmitted symbionts that infect the bacteriocytes of the cockroach *Blattella germanica*, migrate from somatic tissues within their bacteriocytes which are subsequently incorporated into the ovary, from which the bacteria can go on to infect developing oocytes [46], [47], suggesting that this may also applicable to some *Wolbachia*-insect systems. Third, *Wolbachia* may have specialised on particular somatic tissues to produce effects other than those associated with sex ratio distortion [3]. A similar pattern of high infection intensity in fat body and other somatic tissues was seen in the mosquito *Anopheles gambiae* after transfection with the virulent popcorn *Wolbachia* strain wMelPop, where in fact infection of the reproductive tissues did not occur [48]. The fat body is a nutritionally rich environment that supports obligate symbionts in many other insects [49], and it may therefore be a good place for *Wolbachia* to proliferate to high numbers. The fat body and haemolymph are integral to the regulation of the insect immune system [50]. Modulation of the immune response by *Wolbachia* has been noted with increased immune functions being found with infection in some insect species [24], [51], and decreased immune responses found in others [15], [23]. The high intensity of *Wolbachia* in the fat body and haemolymph seen here could therefore suggest that the tissue tropism may be an adaptation of *Wolbachia* to modulate the host immune system.

It is now becoming clear that horizontal transmission of *Wolbachia* can occur frequently [16], [19], [44]. To date experimental evidence has only been found for horizontal transmission between parasitoids and their hosts [19]–[21], but transmission by predation or a shared food resource has also been implicated by the effects of host ecology on *Wolbachia* strain distribution [4], [10], [52], [53]. The high intensity of *Wolbachia* in the haemolymph found here could enhance transmission by either parasitoids or via blood contact. The experimentally demonstrated horizontal transmission of *Wolbachia* to parasitoids from their host, and in fact all of the horizontal transmission routes suggested for *Wolbachia*, require ingested *Wolbachia* whether from host or food, to cross the gut wall to establish an infection. In a paper examining the change in *Wolbachia* intensity over the lifetime of ant workers, relatively high loads of *Wolbachia* were found extracellularly in the gut and faeces [25], supporting the significant levels of *Wolbachia* found in tissues in this study. It has been suggested that this may point to a nutritional role for the *Wolbachia* infecting these ants [25]. We find higher levels of infection in other tissues suggesting that other phenotypic effects may be important and that high *Wolbachia* intensities in the gut and faeces may be more important in terms of transmission. The demonstration that *Wolbachia* can cross tissues within a host [54], makes it not unreasonable that it could be excreted into the gut lumen and the relatively high levels of *Wolbachia* in faeces corroborate this. *Wolbachia* can survive in cell free media for up to a week without a reduction in viability [55], and so it is possible that the *Wolbachia* in faeces may be viable and able to transmit via the faecal-oral route. Faecal-oral transmission has particular potential in social insects because of the high population density in their colonies and the fact that many species engage in stomodeal or proctodeal trophallaxis [56]. This potential is perhaps even greater in leaf-cutting ants because they manure their food, with their own faeces [57]. Recent work suggests that feeding habitats on mushrooms are important for the transmission of *Wolbachia* [10], and the *Erhlichia* and *Rikettsia* bacteria which are sister to *Wolbachia* can both be transmitted via the contamination of broken skin with arthropod faeces [58], providing further support that such a transmission route is possible.

This study suggests that somatic tissues and sterile hosts may not be the evolutionary dead-end for *Wolbachia* that is commonly thought. *Wolbachia* is typically thought of as an intracellular, sex ratio-distorting symbiont which transmits exclusively from mother to offspring, but our results add to a growing body of evidence which suggests that its effects and transmission may be more diverse. Similar investigations of tissue-tropism in other symbionts are likely to prove informative in better understanding the complexities of host-symbiont interactions.

## Supporting Information

Figure S1 **Colony specific** ***Wolbachia*** **infection intensities.** Comparison of mean ± s.e. relative *Wolbachia* intensities (RQ) in fat body, hindgut, haemolymph and ovary/midgut of *Acromyrmex echinatior* leaf-cutting ant workers and queens from four colonies, and worker faeces from three colonies. Sample size is shown above each corresponding bar. Relative *Wolbachia* intensity refers to the intensity of *Wolbachia* normalized against the host 18S control gene.
(TIF)

Table S1 **qPCR assay sequences.** Primer and probe sequences for host and *Wolbachia* qPCR assays.
(TIF)
